# Assessing the impact of posture on brain volume in healthy subjects with a rotatable cryogen-free 1.5T superconducting MRI

**DOI:** 10.3389/fnins.2025.1644236

**Published:** 2025-09-02

**Authors:** Shiying Ke, Yulin Wang, Jichang Zhang, Jie Zeng, Shengyang Niu, Jianjun Zheng, Thomas Meersmann, Chengbo Wang

**Affiliations:** ^1^Department of Electrical and Electronic Engineering, Faculty of Science and Engineering, University of Nottingham Ningbo China, Ningbo, China; ^2^Department of Medical Imaging, Quanzhou Medical College, Quanzhou, China; ^3^Xingaoyi Medical Equipment Company, Ltd., Ningbo, China; ^4^Department of Radiology, Ningbo No. 2 Hospital, Ningbo, China; ^5^School of Medicine, Sir Peter Mansfield Imaging Centre, Translational Medical Sciences, University of Nottingham, Nottingham, United Kingdom; ^6^NIHR Nottingham Biomedical Research Centre, Nottingham University Hospitals NHS Trust, Queen’s Medical Centre, Nottingham, United Kingdom; ^7^Department for Strategic Development of Health Science and Technology, University of Nottingham Ningbo China, Ningbo, Zhejiang, China

**Keywords:** MRI, brain structures, volumetric differences, postural effects, MP-RAGE

## Abstract

**Background:**

Magnetic Resonance Imaging (MRI) is crucial for detailed visualization of brain structure and function. However, conventional supine imaging limits the exploration of how posture impacts brain morphology. While recent advancements in upright MRI systems have enabled studies of postural effects on various body systems, investigations into posture’s impact on brain anatomy remain limited.

**Method:**

This study investigated volumetric differences between upright and supine positions to establish a baseline for future investigations into how posture influenced brain structure. Thirty-one healthy volunteers underwent scans using a rotatable cryogen-free 1.5T MRI scanner in supine and upright postures. The 3D T1-weighted MP-RAGE brain images were segmented into 109 regions, and volume changes across these regions were analyzed.

**Result:**

Volumetric analysis across 109 brain regions in both supine and upright postures shows minimal changes, with most regions displaying variations within a ±5% range. The coefficient of variation (COV) indicated that posture-induced volume changes are even smaller than the measurement precision of the method. These findings provide a solid groundwork for future studies on the effects of posture on brain structure.

**Conclusion:**

The majority of brain regions exhibited no significant volumetric differences between supine and upright positions, suggesting that brain structure remains consistent and stable across different postures. These findings offer valuable insights for future research on the postural influences on brain morphology.

## 1 Introduction

Magnetic Resonance Imaging (MRI) has emerged as a crucial tool for visualizing brain structures and functions due to its non-invasive nature, high spatial resolution, and ability to provide detailed anatomical and functional insights (Thompson et al., 2005).

Traditionally, most MRI scans are conducted with subjects in a supine position, a practice necessitated by the design constraints of conventional MRI scanners (Eşer et al., 2007). Although effective for many diagnostic purposes, this fixed, non-physiological posture may not fully represent how brain structures and functions operate, as human bodies are typically more dynamic and engage in various postures in daily life (Alperin et al., 2005a). Posture is known to significantly influence physiological and anatomical characteristics across the body. For example, shifting from lying down standing can alter spinal alignment, change lung capacity, and modify cardiovascular dynamics, among other effects (Rossberg and Peňaz, 1988; Fawcett et al., 2021). These observations underscore the importance of incorporating different postures in studies of body systems and their functions. However, traditional MRI systems are limited in their ability to assess posture-related structural differences, highlighting the need for innovative multi-positional MRI technologies.

To overcome these limitations, researchers have introduced various MRI systems capable for upright or weight-bearing imaging, including Fonar’s 0.6T system (Nicholson et al., 2023), Siemens’ 1.0T system (Gufler et al., 2004), and G-Scan’s 0.25T system (Lee et al., 2015b), et al. Although these systems operate at relatively low magnetic field strengths, which may reduce image resolution and signal-to-noise ratio (SNR) during scanning (Marques et al., 2019), they have proven the research value in the effects of specific postures on body structures (Shymon et al., 2014; Hansen et al., 2019). The 1.5T MRI system provides higher SNR and spatial resolution, allowing for clearer visualization of small lesions and anatomical structures, thus enhancing the quantitative analysis capabilities of the images and assisting clinicians in making more accurate diagnostic and treatment decisions (Magee et al., 2003; Arnold et al., 2023; Schmid et al., 1999). For instance, upright MRI has proven notably effective in detecting spinal stenosis (Fawcett et al., 2021) and in visualizing medial meniscus extrusion in the knee under weight-bearing conditions (Draper et al., 2011). Additionally, these systems offer significant benefits in the early diagnosis of pelvic organ prolapse (Shaikh et al., 2021), thereby facilitating more prompt and effective treatment. Despite significant technological advancements, most existing studies have focused on other body systems, leaving research on the impact of posture on brain morphology relatively underexplored. Regions of the brain, such as the pituitary gland, cerebellum, and choroid plexus, which are more susceptible to gravitational effects, are particularly affected. A previous study has shown that the choroid plexus undergoes morphological changes under the influence of gravity (Wang et al., 2025). These findings highlight the gap in our understanding of how posture affects brain structure and emphasize the pressing need for innovative MRI systems specifically designed for brain research.

While traditional MRI systems and some newly developed upright MRI systems have significantly advanced our understanding of postural effects on other body systems, research in the brain domain remains limited due to technical and methodological challenges. In prior studies, the evaluation of brain regions in the upright posture was typically done through visual assessment by two blinded clinicians, without image segmentation. This approach likely arose from the lack of automatic segmentation technology available in 2001, and the image quality at that time may not have met the requirements for accurate segmentation (Nakada and Tasaka, 2001). Additionally, there was relatively limited research on upright head imaging. To address these challenges, we have developed a cryogen-free 1.5T superconductive MRI system that can operate with an active magnetic field during scanner rotation (Wang et al., 2023). This system offers a unique opportunity to study the impact of different postures on brain morphology under conditions that closely mimic everyday physiological activities. This innovative design allows for high-resolution imaging in both supine and upright positions without compromising image quality.

The objective of this study is to investigate and validate whether significant volumetric changes occur in various brain regions between upright and supine positions in healthy individuals. By identifying and quantifying these differences, we aim to elucidate the potential clinical implications of postural effects on brain structure. These findings could offer valuable insights for advancing clinical brain research, especially in conditions where postural influences may play a significant role.

## 2 Method

**Figure 1:**
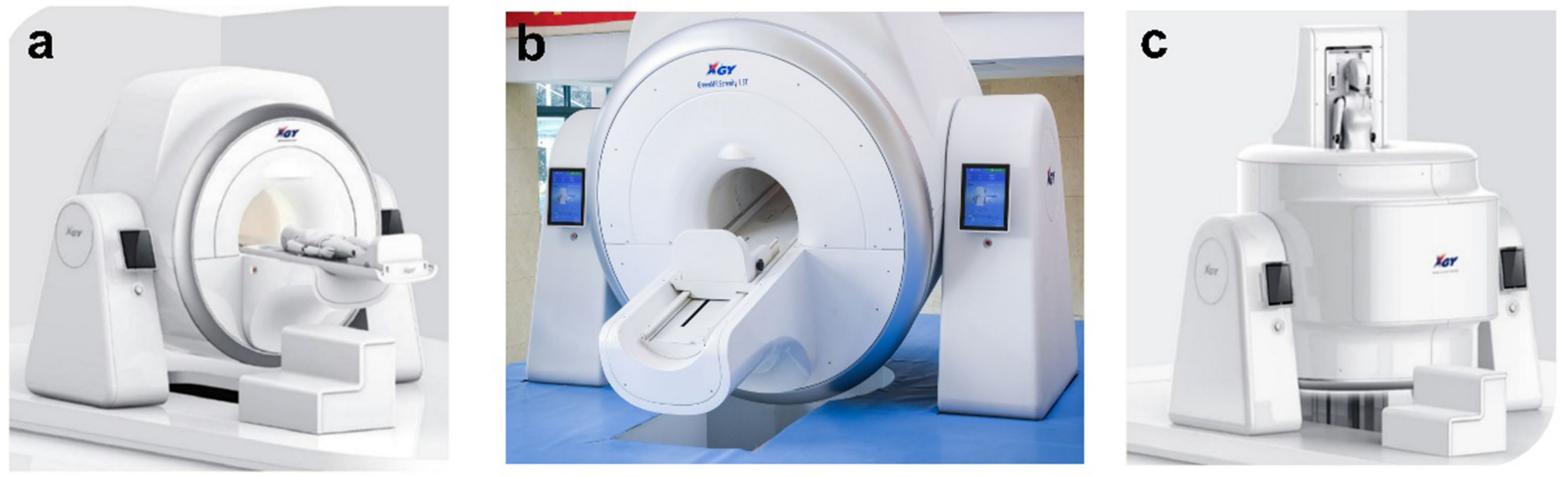
Design and configuration of the 1.5T superconductive cryogen-free spin MRI system: **(a)** at 0° angle for supine scanning, **(b)** at 45° angle for an oblique view, and **(c)** at 90° angle for upright scanning.

All imaging was conducted using a state-of-the-art, rotatable, cryogen-free superconductive 1.5T MRI scanner (XGY-Spin MRI-R001, XGY, Ningbo, China), which served as the primary imaging platform (Figure 1). This prospective study was carried out between August to September 2024, with ethical approval granted by the institutional ethics committees of both the University of Nottingham Ningbo China, and Ningbo No. 2 Hospital. Written informed consent was obtained from all participants, ensuring compliance with established guidelines for human research. Additionally, participant confidentiality was meticulously maintained by anonymizing data prior to analysis.

The inclusion criteria for eligible participants were aged 20–35 years, without any history of neurological or psychiatric disorders, and met all MRI scanning safety requirements. The exclusion criteria included significant brain abnormalities, a history of brain surgery, systemic diseases affecting brain structure (e.g., hypertension or diabetes), or pregnancy. Initially, 33 individuals were enrolled; however, one was excluded due to the presence of a cerebral lipoma. Additionally, due to a computer system error, data from one individual was corrupted, leading to exclusion. As a result, the final cohort consisted of 31 healthy volunteers (mean age: 26.5 ± 3.7 years; comprising 16 males and 15 females). Each subject underwent an MRI scan using a 3D T1-weighted MPRAGE sequence, with the following settings: Repetition Time (TR) = 10.0 ms, Echo Time (TE) = 3.4 ms, Field of View (FOV) = 230 mm × 230 mm × 187.2 mm, Sampling Matrix = 192 × 192 × 156, Reconstruction Matrix = 480 × 480 × 156, Spatial Resolution = 0.48 mm × 0.48 mm × 1.20 mm, Slice Thickness = 1.20 mm, and Flip Angle = 12°. The scan time for the 3D MP-RAGE sequence was 4 min and 15 s. Scans were performed in two different postures—supine (0 degrees) and upright (90 degrees). For supine scans, participants’ heads were supported by foam pads to minimize motion. In the upright posture, restraint straps and additional foam pads were used to secure and align the head, effectively reducing motion artifacts.

**Figure 2:**
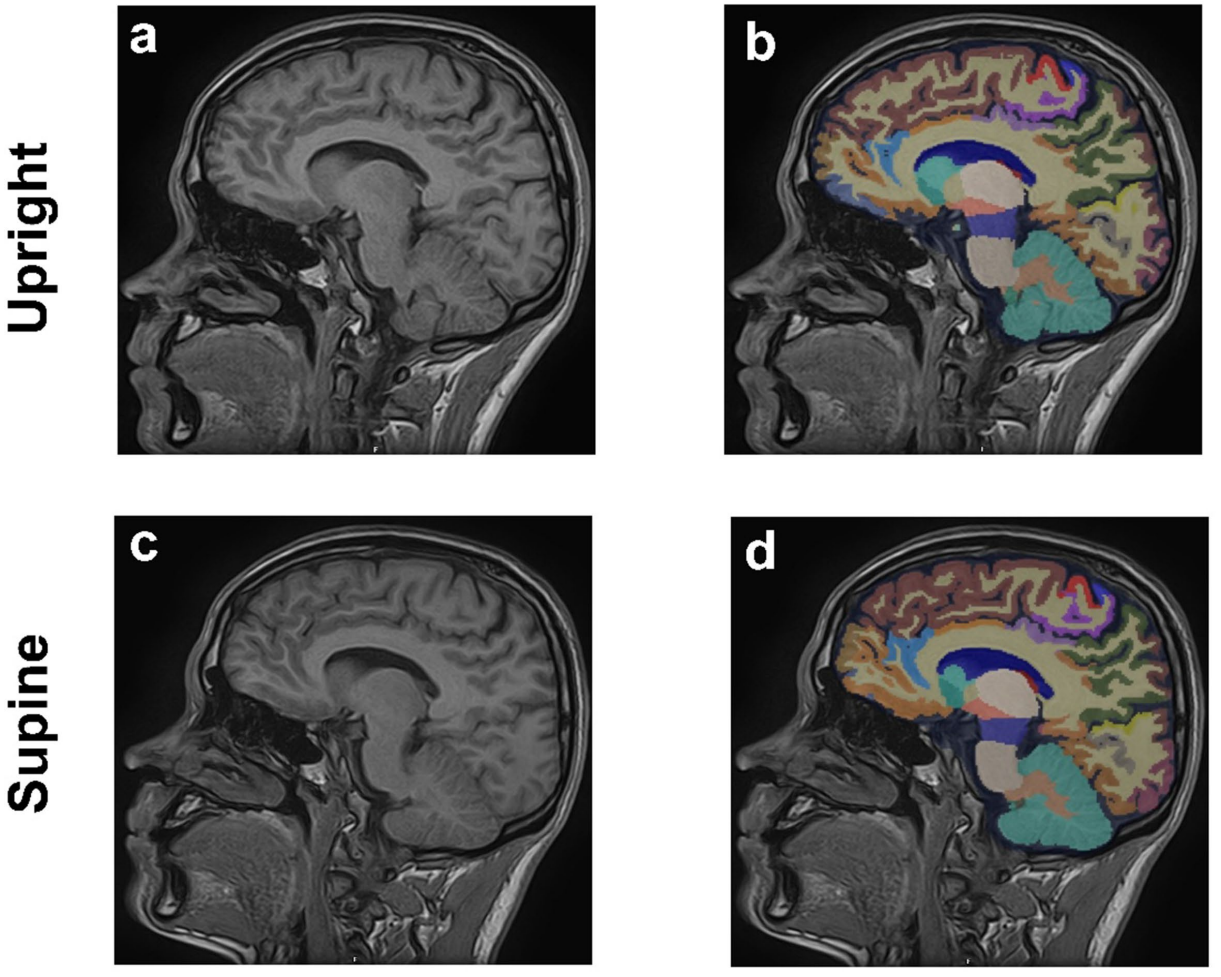
Sagittal 3D T1-weighted MPRAGE MRI images of the brain from a 29-year-old male in upright **(a,b)** and supine **(c,d)** positions. Anatomical images are shown in **(a,c)**, **(b,d)** corresponding regional brain segmentations.

A comprehensive volumetric analysis of 109 brain regions was performed using the United Imaging analysis system to assess structural changes between the two postures. The automated whole-brain segmentation from 3D T1-weighted MPRAGE images was conducted via the uAI Research Portal (United Imaging Intelligence, China) (Wu et al., 2023), a clinical research platform developed using Python (version 3.7.3). This process utilized the PyRadiomics package^1^ (Liu et al., 2022), enabling precise segmentation of the whole brain into 109 distinct regions and providing volumetric measurements for each region to assess structural changes between the two postures (Figure 2).

To assess both the reproducibility and repeatability of this analysis method, an experiment was conducted with three selected subjects. Measurements of brain volume in the supine posture were performed for each subject at three time points: Day 1 (first scan), Day 30 (second scan, initial scan), and Day 30 (third scan, taken 30 min after the second scan). This approach aimed to determine the stability of measurements over time and the reliability of the method. The coefficient of variation (COV) (Bland and Altman, 2010) was calculated using the formula: COV = (Standard deviation/Mean) × 100% (Abdi, 2010). And the average COV was calculated across the three subjects. In this study, the COV was expressed as a percentage, providing a depiction of measurement variability and aiding in the assessment of the precision and consistency of the measurements.

Subsequently, brain volume was measured in both the supine and upright positions for all 31 subjects to evaluate changes in brain region volumes between these two postures. Additionally, the percentage change in brain volume between the upright and supine measurements was calculated using the formula: (V_upright_ − V_supine_)/(V_upright_) × 100%, where V_supine_ and V_upright_ represent the brain volume in the supine and upright postures, respectively. To ensure the validity of the parametric analysis, the Shapiro–Wilk test was conducted for each brain region to assess whether the volume data conformed to a normal distribution. Only regions that satisfied the normality assumption were included in the subsequent two-tailed paired Student’s t-test. For regions that did not meet the normality assumption, the non-parametric Wilcoxon signed-rank test was applied instead. These statistical tests were used to evaluate volume differences between the two positions. Multiple comparison corrections were also applied to control for Type 1 errors.

## 3 Results

Table 1 presents the demographic characteristics of the participants, all of whom are young adults. This selection minimizes the influence of age-related brain volume decline. Additionally, the balanced gender ratio helps reduce the potential impact of sex differences on brain structure.

**Table 1:** Demographic characteristics of the study participants.

Normal volunteers	Pre-scan	Post-scan	*p* value
Age (years)	26.5 ± 3.7		
Gender (M/F)	16/15		
Height (cm)	168.8 ± 6.6		
Weight (kg)	64.8 ± 11.7		
HR (beats/min)	78.9 ± 13.8	76.7 ± 11.6	0.501
Systolic BP (mmHg)	115.2 ± 12.6	110.5 ± 11.7	0.148
Diastolic BP (mmHg)	74.1 ± 7.2	70.9 ± 7.5	0.101

HR, heart rate; BP, blood pressure.

Imaging was conducted on all subjects using the spin MRI, with no adverse effects. Figure 2 illustrates the segmentation of 109 distinct brain regions from 3D T1-weighted MPRAGE images of a sample subject in both upright and supine positions, performed using the uAI Research Portal. Volumetric measurements for each region were obtained to evaluate structural differences between the two positions.

**Figure 3:**
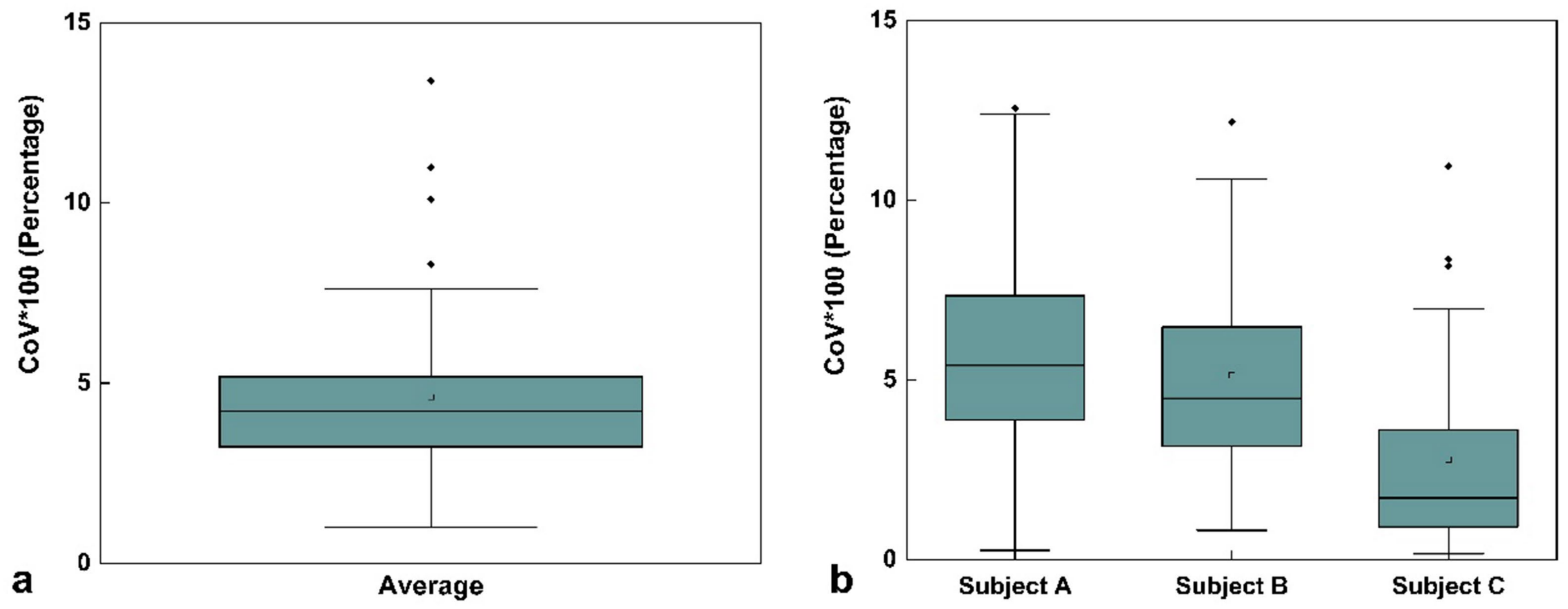
Box plot of mean coefficient of variation (COV) values for volume measurements across three time points: first scan on Day 1, second scan on Day 30 (initial scan), and third scan on Day 30 (30 min after the second scan) for three selected subjects in the same supine posture. **(a)** COV values averaged across all three subjects. **(b)** COV values for each individual subject (Subject A, Subject B, and Subject C) with three repeated scans. The square markers represent the mean values. Data represent the COV values across all 109 brain regions.

As shown in Figure 3, the average COV across the three subjects was 4.61% ± 2.60%, indicating good measurement repeatability. The COV values remained consistent across the three time points: Day 1 (first scan), Day 30 (second scan, initial scan), and Day 30 (third scan, conducted 30 min after the second scan). The absence of significant fluctuation further suggests that the scanning method provides reliable precision and temporal stability. Overall, the low mean COV reflects the high repeatability and low variability of the brain volume measurements in the supine posture.

To further assess the measurement precision of the scanning method, we calculated the coefficient of variation (COV) for brain volume measurements in three subjects across the three designated time points: Day 1 (first scan), Day 30 (second scan, initial scan), and Day 30 (third scan, conducted 30 min after the second scan). For each subject, the COV was determined by dividing the standard deviation of the three volume measurements by their mean (Figure 3b). The final mean COV was obtained by averaging the individual COV values across the three subjects (Figure 3a).

**Figure 4:**
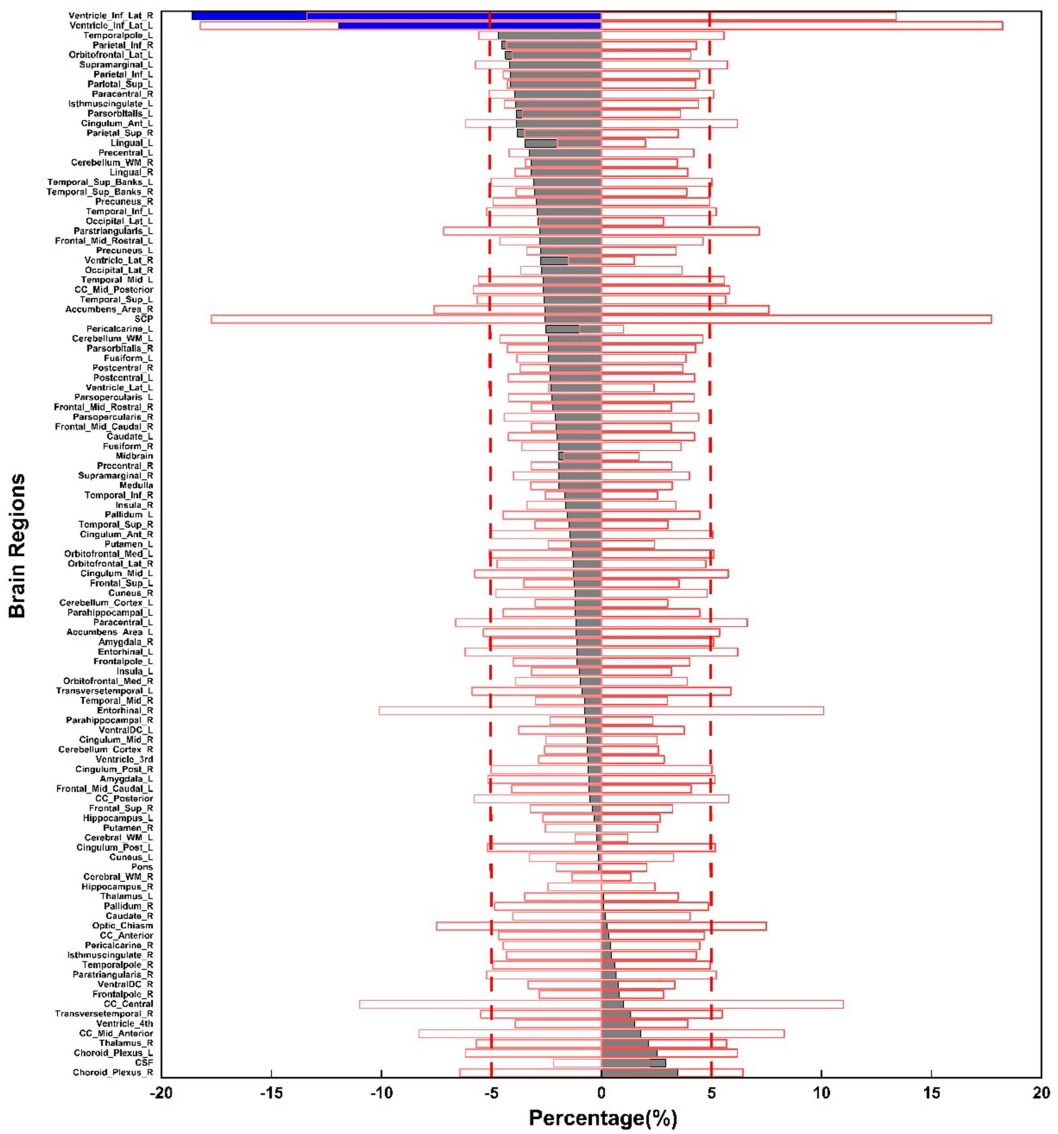
Percentage volume changes between upright and supine postures across 109 brain regions, illustrated in the gray and blue bars. The red box displays the mean COV values for volume measurements across three selected subjects at three time points.

Figure 4 shows the overlap of the coefficient of variation (COV) values with the percentage volume changes across 109 brain regions between upright and supine postures. The gray bars represent brain regions with volume changes within the ±5% range, while the blue bars indicate regions with volume changes exceeding 5%. Most brain regions exhibit volume changes within the ±5% range, with only a few regions exceeding this threshold, suggesting that the volume changes between the two postures are very stable for the majority of brain regions.

Figure 4 illustrates that most of the gray bars, representing the average postural volume changes across the 31 subjects, fall within the red box, which denotes the average COV across the three selected subjects for each segmented brain region. This suggests that most brain regions are highly stable, with their postural changes resembling those observed in the same supine posture across different time points.

**Figure 5:**
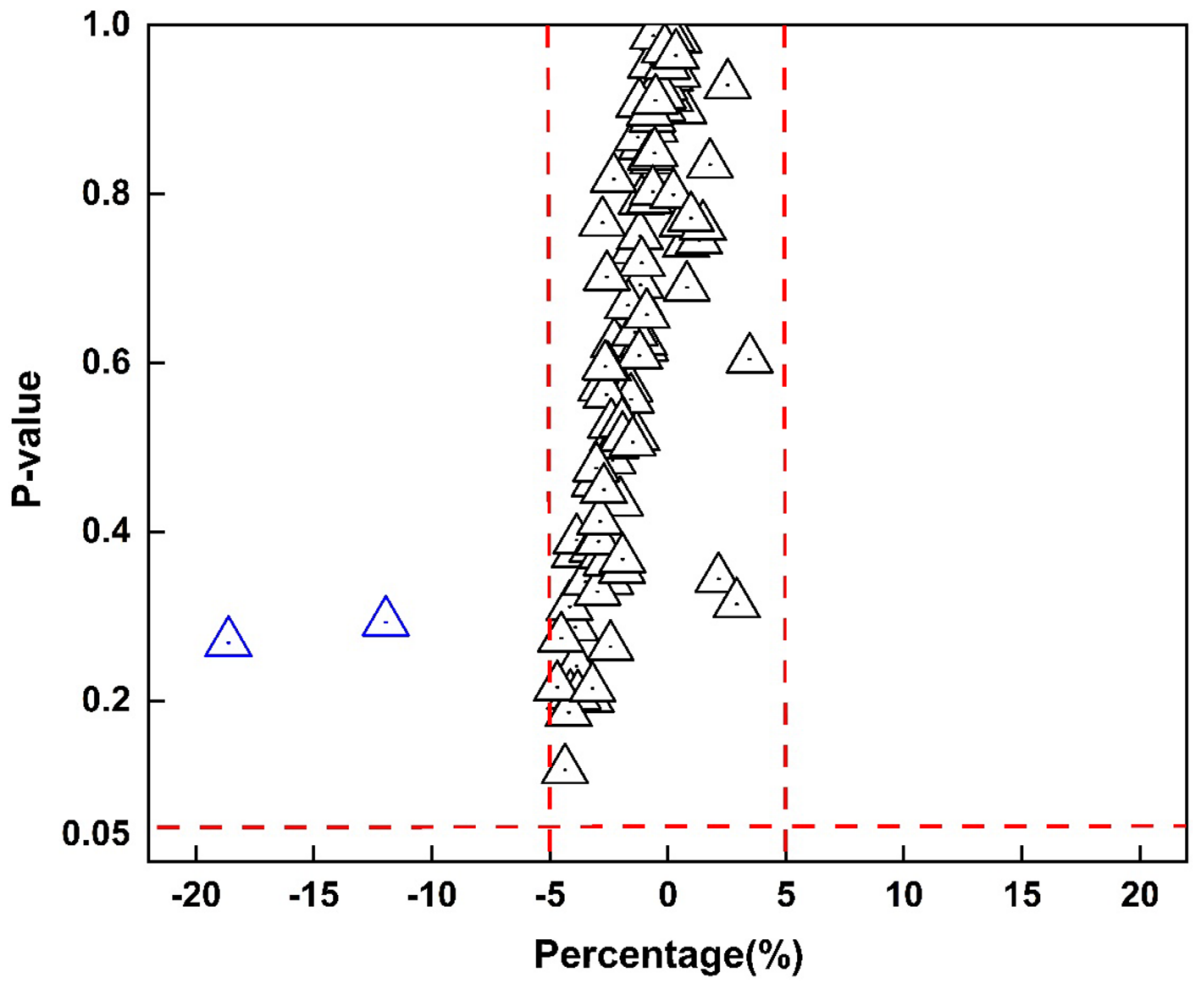
Scatter plot of percentage volume change versus *p*-values for 109 segmented brain regions. The x-axis represents the percentage change, while the y-axis shows p-values. Red dashed lines mark ±5% on the x-axis and a significance level of 0.05 on the y-axis, where each triangle represents a brain region.

Figure 5 presents a scatter plot illustrating the relationship between percentage volume changes and *p*-values. The majority of brain regions display minimal volume changes, with *p*-values approaching 1, whereas a few regions near the threshold of significance with *p*-values close to 0.05, further underscoring the overall stability of brain volumes. Multiple comparison corrections were also applied, and the results remained consistent, reinforcing the stability and robustness of the observed findings.

**Figure 6:**
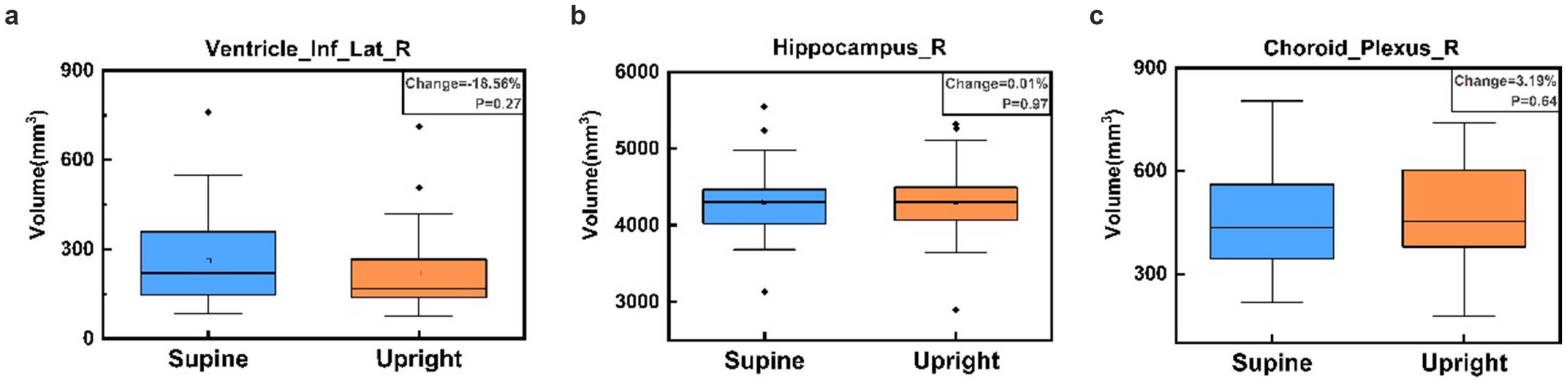
Box plots comparing brain region volumes between supine (blue) and upright (orange) postures for the Right Inferior Lateral Ventricle **(a)**, Right Hippocampus **(b)**, and Right Choroid Plexus **(c)**. Panel **(a)** illustrates the largest negative percentage change in the Right Inferior Lateral Ventricle, panel **(b)** shows the volume change closest to zero in the Right Hippocampus, and panel **(c)** displays the largest positive percentage change in the Right Choroid Plexus. No significant volume differences are observed between postures for these regions, as indicated by the p-values in each panel.

Most brain regions exhibited minimal volume changes between upright and supine postures. Notably, there was no significant difference in intracranial volume (ICV) between the two positions: supine position ICV was 1407981.60 ± 138923.61 mm^3^, and upright position ICV was 1402848.68 ± 135176.12 mm^3^ (*p* = 0.89). Figure 6a demonstrates that the Inferior Lateral Ventricle showed the most substantial negative volume change (−18.56%), which exceeded the ±5% threshold; however, this difference was not statistically significant (*p* = 0.27). In contrast, the hippocampus depicted in Figure 6b showed a negligible volume change of 0.01% (*p* = 0.97), exemplifying the typical stability observed across most brain regions. Figure 6c highlights the Choroid Plexus, which showed the largest positive volume change at 3.19% (*p* = 0.64), yet maintained a relatively stable distribution.

## 4 Discussion

To the best of our knowledge, this study is the first to employ a cryogen-free, rotatable superconductive MRI system in the investigation of brain volume changes. While the system is capable of maintaining a continuous magnetic field during scanner rotation, it is important to note that all scans in this study were conducted at fixed positions (either 0 or 90 degrees), rather than during continuous rotation. We investigated the effects of postural changes on brain volume in healthy individuals by comparing supine and upright positions using this innovative rotatable MRI scanner, quantifying the statistical differences between these two postures. Our results indicate that postural changes have a minimal impact on brain volume in healthy individuals, which supports existing knowledge about the high stability of brain structure.

Specifically, the volume changes in most brain regions between the upright and supine positions were within a ±5% range, with the majority of regions showing near-zero volume changes and *p*-values close to 1, further validity the stability and reliability of the brain structure. This is consistent with prior upright MRI studies, which have shown that gravity exerts a negligible effect on brain structure, even in conditions such as brain atrophy (Nakada and Tasaka, 2001). However, as illustrated in Figure 6a, the inferior lateral ventricle, where CSF is contained and circulated through, showed a positive shift in volume from upright to supine, yet the overall variability remained low. This region, adjacent to cerebrospinal fluid (CSF)-filled areas, has been documented in prior research to experience a significant increase in CSF flow when supine, suggesting that these regions may be more susceptible to postural changes and may exhibit unique anatomical or hemodynamic characteristics (Muccio et al., 2021; Alperin et al., 2015). Previous studies have reported that CSF flow, velocity, and stroke volume decrease significantly in the upright posture, with these changes closely related to body position (Yukun et al., 2025). Although our study did not include direct CSF flow measurements, the volumetric trend observed may reflect posture-dependent redistribution of CSF. These findings provide preliminary structural evidence that may contribute to understanding how posture influences CSF dynamics.

Further investigations integrating structural, functional, and flow-sensitive imaging modalities are warranted to elucidate the mechanisms by which posture influences CSF distribution and ventricular morphology.

Furthermore, previous studies have demonstrated that, compared to the supine position, the upright posture reduces CSF oscillatory volume by approximately 48%, significantly increases intracranial compliance (ICC), and decreases intracranial pressure (ICP) by approximately 2.4 times (Alperin et al., 2005b). These findings highlights the dynamic role of CSF in regulating intracranial pressure across different postural orientations, effectively maintaining intracranial pressure equilibrium. Notably, prior research has also shown that, even in cases of cerebrospinal fluid leakage, brain imaging differences between the upright and supine positions remain insignificant (Schievink and Tourje, 2007). This further underscores that, despite the significant effects of upright posture on CSF distribution and ICP, the brain’s intrinsic regulatory mechanisms ensure the stability of volume and morphology across brain regions (Lee et al., 2015a).

On the other hand, the hippocampus (Figure 6b) demonstrated the overall stability of most brain regions, with minimal variation between postures. Likewise, despite relatively larger volume changes in the choroid plexus, its low variability further highlights the consistency of measurements across different postural conditions.

The results depicted in Figure 3 demonstrate the good precision and repeatability of the current method. Measurement values for all three subjects exhibited minimal fluctuation across different time points indicating that the method provides consistent volume measurements. By calculating COV, we found that the measurement consistency for the vast majority of brain regions is high, indicating that the current measurement method demonstrates good repeatability and stability. The volume changes in brain regions induced by posture shifts mostly fall within the measurement error range, making them undetectable by the current method.

## 5 Limitation

A limitation of this study is that the COV measurements were derived from data obtained from only three subjects. Such a small sample size may not be sufficiently robust to provide a comprehensive evaluation of the precision and consistency of the method at this scale. Although the results indicate that the method operates effectively within a reasonable margin, the limited sample size could introduce variability in the accuracy of the measurements. To achieve a more reliable estimation of the COV, it would be ideal to conduct multiple scans with a larger cohort, each undergoing at least three separate measurements. However, implementing such a protocol would significantly extend the required scanning time and necessitate multiple visits from the volunteers, which presents considerable logistical challenges. Consequently, the COV values observed in this study may not fully reflect the true precision of the method, highlighting the need for further studies involving larger sample sizes and repeated scans to confirm the reliability and generalizability of the findings.

Another limitation of this study concerns the accuracy of choroid plexus segmentation. Although we utilized the PyRadiomics package for segmentation of 109 brain regions, the tool is based on FreeSurfer, which has shown limited performance in choroid plexus segmentation (Zhao et al., 2020). As the choroid plexus is a critical structure influenced by body position and CSF flow, previous studies have demonstrated that deep learning-based approaches perform significantly better in this context (Yazdan-Panah et al., 2023; Eisma et al., 2024; Zhao et al., 2020). However, due to the scope of this study, these advanced deep learning techniques were not implemented. It is worth noting that a parallel study using this rotatable MRI system has analyzed the choroid plexus and provides supplementary data (Wang et al., 2025). We acknowledge that the segmentation method used in this study may not provide the same level of precision as newer techniques, which may affect the reliability of the volumetric findings related to the choroid plexus. In future work, we plan to explore these advanced deep learning techniques to improve segmentation accuracy.

## 6 Conclusion

In conclusion, while most brain regions showed no significant differences between supine and upright positions, these findings deepen our understanding of the potential clinical implications of postural effects on brain structure and provide valuable insights for future studies in clinical brain research.

## Data Availability

The datasets presented in this article are not readily available because this prospective study was approved by the institutional ethics committees of both the University of Nottingham Ningbo China and Ningbo No. 2 Hospital. Participant confidentiality was carefully maintained by anonymizing the data prior to analysis. Due to sensitivity concerns, the data supporting the findings of this study are not openly available. Access to the data may require institutional or ethical approval due to privacy considerations. Further, the data are available from the corresponding author upon reasonable request. Requests to access the datasets should be directed to chengbo.wang@nottingham.edu.cn.

## Footnotes

^1^ https://pyradiomics.readthedocs.io

## Author’s note

Parts of this work have been presented as a digital poster at the 33rd Annual Meeting of the International Society for Magnetic Resonance in Medicine (ISMRM), 2025.

## Glossary

Precentral_L: Left Precentral Gyrus
Precentral_R: Right Precentral Gyrus
Postcentral_L: Left Postcentral Gyrus
Postcentral_R: Right Postcentral Gyrus
Paracentral_L: Left Paracentral Lobule
Paracentral_R: Right Paracentral Lobule
Frontal_Sup_L: Left Superior Frontal Gyrus
Frontal_Sup_R: Right Superior Frontal Gyrus
Frontal_Mid_Rostral_L: Left Rostral part of the Middle Frontal Gyrus
Frontal_Mid_Rostral_R: Right Rostral part of the Middle Frontal Gyrus
Frontal_Mid_Caudal_L: Left Caudal part of the Middle Frontal Gyrus
Frontal_Mid_Caudal_R: Right Caudal part of the Middle Frontal Gyrus
Frontalpole_L: Left Frontal Pole
Frontalpole_R: Right Frontal Pole
Orbitofrontal_Lat_L: Left Lateral Orbitofrontal Cortex
Orbitofrontal_Lat_R: Right Lateral Orbitofrontal Cortex
Orbitofrontal_Med_L: Left Medial Orbitofrontal Cortex
Orbitofrontal_Med_R: Right Medial Orbitofrontal Cortex
Parsopercularis_L: Left Pars Opercularis
Parsopercularis_R: Right Pars Opercularis
Parsorbitalis_L: Left Pars Orbitalis
Parsorbitalis_R: Right Pars Orbitalis
Parstriangularis_L: Left Pars Triangularis
Parstriangularis_R: Right Pars Triangularis
Insula_L: Left Insula
Insula_R: Right Insula
Cingulum_Ant_L: Left Anterior Cingulum
Cingulum_Ant_R: Right Anterior Cingulum
Cingulum_Mid_L: Left Middle Cingulum
Cingulum_Mid_R: Right Middle Cingulum
Cingulum_Post_L: Left Posterior Cingulum
Cingulum_Post_R: Right Posterior Cingulum
Isthmuscingulate_L: Left Isthmus of Cingulate Cortex
Isthmuscingulate_R: Right Isthmus of Cingulate Cortex
Hippocampus_L: Left Hippocampus
Hippocampus_R: Right Hippocampus
Parahippocampal_L: Left Parahippocampal Gyrus
Parahippocampal_R: Right Parahippocampal Gyrus
Amygdala_L: Left Amygdala
Amygdala_R: Right Amygdala
Caudate_L: Left Caudate Nucleus
Caudate_R: Right Caudate Nucleus
Putamen_L: Left Putamen
Putamen_R: Right Putamen
Pallidum_L: Left Pallidum
Pallidum_R: Right Pallidum
Thalamus_L: Left Thalamus
Thalamus_R: Right Thalamus
Accumbens_Area_L: Left Nucleus Accumbens
Accumbens_Area_R: Right Nucleus Accumbens
VentralDC_L: Left Ventral Dorsal Cortex
VentralDC_R: Right Ventral Dorsal Cortex
Choroid_Plexus_L: Left Choroid Plexus
Choroid_Plexus_R: Right Choroid Plexus
Ventricle_Lat_L: Left Lateral Ventricle
Ventricle_Lat_R: Right Lateral Ventricle
Ventricle_Inf_Lat_L: Left Inferior Lateral Ventricle
Ventricle_Inf_Lat_R: Right Inferior Lateral Ventricle
Parietal_Sup_L: Left Superior Parietal Lobule
Parietal_Sup_R: Right Superior Parietal Lobule
Parietal_Inf_L: Left Inferior Parietal Lobule
Parietal_Inf_R: Right Inferior Parietal Lobule
Cuneus_L: Left Cuneus
Cuneus_R: Right Cuneus
Entorhinal_L: Left Entorhinal Cortex
Entorhinal_R: Right Entorhinal Cortex
Fusiform_L: Left Fusiform Gyrus
Fusiform_R: Right Fusiform Gyrus
Lingual_L: Left Lingual Gyrus
Lingual_R: Right Lingual Gyrus
Pericalcarine_L: Left Pericalcarine Cortex
Pericalcarine_R: Right Pericalcarine Cortex
Precuneus_L: Left Precuneus
Precuneus_R: Right Precuneus
Supramarginal_L: Left Supramarginal Gyrus
Supramarginal_R: Right Supramarginal Gyrus
Temporal_Sup_L: Left Superior Temporal Gyrus
Temporal_Sup_R: Right Superior Temporal Gyrus
Temporal_Mid_L: Left Middle Temporal Gyrus
Temporal_Mid_R: Right Middle Temporal Gyrus
Temporal_Inf_L: Left Inferior Temporal Gyrus
Temporal_Inf_R: Right Inferior Temporal Gyrus
Temporalpole_L: Left Temporal Pole
Temporalpole_R: Right Temporal Pole
Temporal_Sup_Banks_L: Left Superior Temporal Banks
Temporal_Sup_Banks_R: Right Superior Temporal Banks
Transversetemporal_L: Left Transverse Temporal Gyrus
Transversetemporal_R: Right Transverse Temporal Gyrus
Occipital_Lat_L: Left Lateral Occipital Cortex
Occipital_Lat_R: Right Lateral Occipital Cortex
Cerebral_WM_L: Left Cerebral White Matter
Cerebral_WM_R: Right Cerebral White Matter
Cerebellum_Cortex_L: Left Cerebellar Cortex
Cerebellum_Cortex_R: Right Cerebellar Cortex
Cerebellum_WM_L: Left Cerebellar White Matter
Cerebellum_WM_R: Right Cerebellar White Matter
Ventricle_3rd: Third Ventricle
Ventricle_4th: Fourth Ventricle
Pons: Pons
CSF: Cerebrospinal Fluid
Optic_Chiasm: Optic Chiasm
CC_Anterior: Anterior Corpus Callosum
CC_Mid_Anterior: Mid-Anterior Corpus Callosum
CC_Central: Central Corpus Callosum
CC_Mid_Posterior: Mid-Posterior Corpus Callosum
CC_Posterior: Posterior Corpus Callosum
Midbrain: Midbrain
Medulla: Medulla
SCP: Superior Cerebellar Peduncle

## References

[ref1] Abdi H. (2010). “Coefficient of variation” in Encyclopedia of research design, 2010•utdallas.edu, vol. 1, 169–171.

[ref2] Alperin N. Hushek S. Lee S. Sivaramakrishnan A. Lichtor T. (2005a). Mri study of cerebral blood flow and CSF flow dynamics in an upright posture: The effect of posture on the intracranial compliance and pressure. Intracranial pressure and brain monitoring XII. Acta Neurochirurgica-supplementum then supplement-Wien: Springer, 177–181. 10.1007/3-211-32318-x_38 16463846

[ref3] Alperin N. Lee S. H. Bagci A. M. (2015). MRI measurements of intracranial pressure in the upright posture: the effect of the hydrostatic pressure gradient. J. Magn. Reson. Imaging 42, 1158–1163. doi: 10.1002/jmri.24882, PMID: 25753157

[ref4] Alperin N. Lee S. H. Sivaramakrishnan A. Hushek S. G. (2005b). Quantifying the effect of posture on intracranial physiology in humans by MRI flow studies. J. Magn. Reson. Imaging 22, 591–596. doi: 10.1002/jmri.20427, PMID: 16217773

[ref5] Arnold T. C. Freeman C. W. Litt B. Stein J. M. (2023). Low-field MRI: clinical promise and challenges. J. Magn. Reson. Imaging 57, 25–44. doi: 10.1002/jmri.28408, PMID: 36120962 PMC9771987

[ref6] Bland J. M. Altman D. G. (2010). Statistical methods for assessing agreement between two methods of clinical measurement. Int. J. Nurs. Stud. 47, 931–936. doi: 10.1016/j.ijnurstu.2009.10.001 2868172

[ref7] Draper C. E. Besier T. F. Fredericson M. Santos J. M. Beaupre G. S. Delp S. L.. (2011). Differences in patellofemoral kinematics between weight-bearing and non-weight-bearing conditions in patients with patellofemoral pain. J. Orthop. Res. 29, 312–317. doi: 10.1002/jor.21253, PMID: 20949442 PMC5407372

[ref8] Eisma J. J. Mcknight C. D. Hett K. Elenberger J. Han C. J. Song A. K.. (2024). Deep learning segmentation of the choroid plexus from structural magnetic resonance imaging (MRI): validation and normative ranges across the adult lifespan. Fluids Barriers CNS 21:21. doi: 10.1186/s12987-024-00525-9, PMID: 38424598 PMC10903155

[ref9] Eşer İ. Khorshid L. Yapucu Güneş Ü. Demir Y. (2007). The effect of different body positions on blood pressure. J. Clin. Nurs. 16, 137–140. doi: 10.1111/j.1365-2702.2005.01494.x, PMID: 17181675

[ref10] Fawcett L. James S. Botchu R. Martin J. Heneghan N. R. Rushton A. (2021). The influence of spinal position on imaging findings: an observational study of thoracolumbar spine upright MRI in elite gymnasts. Eur. Spine J. 31, 1–8. doi: 10.1007/s00586-021-06997-9 34613494

[ref11] Gufler H. Ohde A. Grau G. Grossmann A. (2004). Colpocystoproctography in the upright and supine positions correlated with dynamic MRI of the pelvic floor. Eur. J. Radiol. 51, 41–47. doi: 10.1016/S0720-048X(03)00133-5, PMID: 15186883

[ref12] Hansen B. B. Nordberg C. L. Hansen P. Bliddal H. Griffith J. F. Fournier G.. (2019). Weight-bearing MRI of the lumbar spine: spinal stenosis and spondylolisthesis. Semin. Musculoskelet. Radiol. 23, 621–633. doi: 10.1055/s-0039-1697937 31745952

[ref13] Lee R. K. Griffith J. F. Leung J. H. Chu W. C. Lam T. Ng B. K.. (2015b). Effect of upright position on tonsillar level in adolescent idiopathic scoliosis. Eur. Radiol. 25, 2397–2402. doi: 10.1007/s00330-015-3597-3, PMID: 25791638

[ref14] Lee H. Xie L. Yu M. Kang H. Feng T. Deane R.. (2015a). The effect of body posture on brain glymphatic transport. J. Neurosci. 35, 11034–11044. doi: 10.1523/JNEUROSCI.1625-15.2015, PMID: 26245965 PMC4524974

[ref15] Liu S. Jie C. Zheng W. Cui J. Wang Z. (2022). Investigation of underlying association between whole brain regions and Alzheimer’s disease: a research based on an artificial intelligence model. Front. Aging Neurosci. 14:872530. doi: 10.3389/fnagi.2022.872530, PMID: 35747447 PMC9211045

[ref16] Magee T. Shapiro M. Williams D. (2003). Comparison of high-field-strength versus low-field-strength MRI of the shoulder. Am. J. Roentgenol. 181, 1211–1215. doi: 10.2214/ajr.181.5.1811211, PMID: 14573405

[ref17] Marques J. P. Simonis F. F. Webb A. G. (2019). Low-field MRI: an MR physics perspective. J. Magn. Reson. Imaging 49, 1528–1542. doi: 10.1002/jmri.26637, PMID: 30637943 PMC6590434

[ref18] Muccio M. Chu D. Minkoff L. Kulkarni N. Damadian B. Damadian R. V.. (2021). Upright versus supine MRI: effects of body position on craniocervical CSF flow. Fluids Barriers CNS 18:61. doi: 10.1186/s12987-021-00296-7, PMID: 34952607 PMC8710028

[ref19] Nakada T. Tasaka N. (2001). Human brain imaging in the upright position. Neurology 57, 1720–1722. doi: 10.1212/WNL.57.9.1720, PMID: 11706122

[ref21] Nicholson L. L. Rao P. J. Lee M. Wong T. M. Cheng R. H. Y. Chan C. (2023). Reference values of four measures of craniocervical stability using upright dynamic magnetic resonance imaging. Radiol. Med. 128, 330–339. doi: 10.1007/s11547-023-01588-8, PMID: 36715785 PMC10020271

[ref22] Rossberg F. Peňaz J. (1988). Initial cardiovascular response on change of posture from squatting to standing. Eur. J. Appl. Physiol. Occup. Physiol. 57, 93–97. doi: 10.1007/BF00691245, PMID: 3342800

[ref23] Schievink W. I. Tourje J. (2007). Upright MRI in spontaneous spinal cerebrospinal fluid leaks and intracranial hypotension. Headache 47, 1345–1346. doi: 10.1111/j.1526-4610.2007.00934.x, PMID: 17927653

[ref24] Schmid M. R. Stucki G. Duewell S. Wildermuth S. Romanowski B. Hodler J. (1999). Changes in cross-sectional measurements of the spinal canal and intervertebral foramina as a function of body position: in vivo studies on an open-configuration MR system. AJR Am. J. Roentgenol. 172, 1095–1102. doi: 10.2214/ajr.172.4.10587155, PMID: 10587155

[ref25] Shaikh N. Zhang H. Brown S. H. Lari H. Lasry O. Street J.. (2021). Synchronous imaging of pelvic geometry and muscle morphometry: a pilot study of pelvic retroversion using upright MRI. Sci. Rep. 11:20127. doi: 10.1038/s41598-021-99305-w, PMID: 34635683 PMC8505414

[ref26] Shymon S. Hargens A. R. Minkoff L. A. Chang D. G. (2014). Body posture and backpack loading: an upright magnetic resonance imaging study of the adult lumbar spine. Eur. Spine J. 23, 1407–1413. doi: 10.1007/s00586-014-3247-5, PMID: 24619606 PMC6339990

[ref27] Thompson P. M. Sowell E. R. Gogtay N. Giedd J. N. Vidal C. N. Hayashi K. M.. (2005). Structural MRI and brain development. Int. Rev. Neurobiol. 67, 285–323. doi: 10.1016/S0074-7742(05)67009-2, PMID: 16291026

[ref28] Wang Y. Ke S. Li J. Zeng J. Zhang J. Niu S.. (2025). Assessing morphological changes in the choroid plexus between standing and supine positions using a rotatable MRI system. Sci. Rep. 15:22329. doi: 10.1038/s41598-025-07985-5, PMID: 40595273 PMC12217260

[ref29] Wang Y. Wang S. Liang P. Brusic V. Zeng J. Liu B.. (2023). Design, construction and performance testing of a 1.5 T cryogen-free low-temperature superconductor whole-body MRI magnet. Supercond. Sci. Technol. 36:045002. doi: 10.1088/1361-6668/acb467

[ref31] Wu J. Xia Y. Wang X. Wei Y. Liu A. Innanje A.. (2023). URP: an integrated research platform for one-stop analysis of medical images. Front. Radiol. 3:1153784. doi: 10.3389/fradi.2023.1153784, PMID: 37492386 PMC10365282

[ref32] Yazdan-Panah A. Schmidt-Mengin M. Ricigliano V. A. Soulier T. Stankoff B. Colliot O. (2023). Automatic segmentation of the choroid plexuses: method and validation in controls and patients with multiple sclerosis. Neuro Image: Clinical 38:103368. doi: 10.1016/j.nicl.2023.103368 PMC10011049 36913908

[ref33] Yukun Z. Liang H. Rongfeng Q. Qi D. Shenyu F. Xin Z.. (2025). Impact of supine and upright positions on cerebral hydrodynamics in healthy subjects: a study using domestic multi-position helium-free MRI. Chin. J. Magn. Reson. Imaging, 1–8. Available online at: https://link.cnki.net/urlid/11.5902.R.20250609.2223.002

[ref34] Zhao L. Feng X. Meyer C. H. Alsop D. C. (2020). Choroid plexus segmentation using optimized 3D U-net. 2020 IEEE 17th international symposium on biomedical imaging (ISBI), IEEE, 381–384.

